# Cybersecurity breaches in medical devices: analyzing FDA safety communications in response to patient security concerns

**DOI:** 10.3389/fdgth.2026.1701551

**Published:** 2026-03-30

**Authors:** Vidya Menon

**Affiliations:** Gujarat National Law University (GNLU), Gandhinagar, Gujarat, India

**Keywords:** cybersecurity breaches, cybersecurity guidances, FDA safety communications, medical devices cybersecurity, patient safety

## Abstract

**Introduction:**

The increasing integration of connected medical devices and internet of things (IoT) technologies in healthcare has significantly improved patient care and operational efficiency. However, this rapid digital transformation has also introduced serious cybersecurity vulnerabilities in medical devices, posing risks to patient safety and sensitive health data. Cybersecurity threats can allow unauthorized remote access to devices, cause device malfunctions, and lead to data breaches. As medical devices become more interconnected within healthcare systems, ensuring their security has become a critical priority for regulators, nanufacturers, and healthcare providers.

**Methods:**

This study examines the cybersecurity safety communications issued by the U.S. Food and Drug Administration (FDA), between 2013 and 2025, using a systematic qualitative content analysis approach. The analysis focuses on identifying the frequency of alerts, the severity of vulnerabilities, and the potential risks posed to healthcare infrastructure and patient safety. The study also reviews regualtory actions and policy frameworks introduced by the FDA to address cybersecurity risks in medical devices.

**Results:**

The analysis found that the FDA issued 18 safety communications related to cybersecurity breaches in medical devices. Among the reported vulnerabilities, 94% were classified as high-risk, indicating severe potential consequences, including unauthorized remote access to medical devices, possible device malfunctions, and exposure of sensitive patient data. Additionally, the results demonstrate a notable increase in FDA cybersecurity safety communications over time, reflecting the growing severity and prevalence of cybersecurity threats in healthcare technologies.

**Discussion:**

The finding emphasize the need for stronger cybersecurity strategies in healthcare. Collaboration among medical device manufacturers, healthcare providers, and regulatory agencies, along with continuous monitoring and regulatory compliance is necessary to protect patient safety and sensitive health data in an increasingly interconnected healthcare environment.

## 1 Introduction

The medical device industry is undergoing transformative changes that are revolutionizing patient care and healthcare delivery. With the increasing reliance on connected medical devices and remote monitoring technologies, patients now have the convenience of tracking their health from home and sharing real-time data with clinicians. Additionally, the introduction of AI algorithms is enhancing operational efficiency by processing large volumes of data, enabling timely interventions, and supporting medical decision-making. As the industry expands, the global market for medical devices is projected to reach $1.30 trillion by 2029, growing at a compound annual growth rate of 9.8% (1).

However, this digital transformation comes with its own set of challenges, particularly in the realm of cybersecurity. The rise of Internet of Things (IoT) devices and remote features in medical technology introduces significant cybersecurity risks. These vulnerabilities can range from patient data breaches to unauthorized access or manipulation of device systems, potentially resulting in catastrophic consequences for patient safety. To address these concerns, the U.S. Food and Drug Administration (FDA) has taken an active role in identifying and reporting cybersecurity vulnerabilities, issuing safety communications to alert healthcare providers and patients, and recommending strategies to mitigate risks (2–4).

While it is impossible to eliminate cybersecurity threats entirely, it is crucial to build robust cybersecurity practices that safeguard patient safety. Despite the FDA's proactive role in addressing these concerns, limited research has been conducted on the impact and effectiveness of their safety communications. Against the backdrop of rising vulnerabilities in the connected medical device industry, this paper aims to analyze cybersecurity breaches reported in medical devices and evaluate the FDA's safety communications in response to patient security concerns from 2013 to 2025. The study will explore the types of vulnerabilities, their impact on patient safety, and the need for stronger cybersecurity standards in medical devices.

The healthcare environment is becoming increasingly complex, requiring collaboration across all stakeholders—manufacturers, healthcare providers, and users—to combat the escalating cybersecurity risks. The FDA's recent initiatives, including issuing guidance documents and the enactment of the Consolidated Appropriations Act, 2023 (“Omnibus”), represent significant steps in ensure the cybersecurity of medical devices. This study will review the FDA safety communications, evaluate their effectiveness, and identify trends, gaps, and challenges in the current regulatory framework, offering recommendations for strengthening future cybersecurity practices in medical device development and deployment.

## 2 Materials and methods

This study employs a systematic qualitative content analysis of all publicly available cybersecurity safety communications and alerts issued by the U.S. Food and Drug Administration (FDA) between June 2013 and January 2025, as published on the official FDA website. This timeframe reflects the entire period during which FDA issued cybersecurity safety communications were available on the FDA website at the time of data collection. Accordingly, the dataset represents the complete set of FDA-issued cybersecurity safety communications available during the study period.

FDA communications were identified through a comprehensive review of the FDA website. Keyword searches using the terms “cybersecurity,” “medical devices,” and “safety communications” were employed solely to locate FDA-issued communications and did not restrict inclusion. All FDA-issued communications that addressed cybersecurity vulnerabilities in medical devices or related systems were included, while communications not issued by the FDA were excluded from the analysis.

The analysis draws on supplementary sources to support interpretation of the risks outlined in each safety alert. These sources include medical device cybersecurity advisories from the Cybersecurity and Infrastructure Security Agency's (CISA), which provide an overview of vulnerabilities, risk assessments, and mitigation strategies, along with recommendations for temporary measures to reduce cybersecurity risks. Additionally, FDA news releases were also reviewed which inform patients of vulnerabilities and offer recommendations to device manufacturers for addressing cybersecurity threats, in line with FDA guidance for pre-market and post-market cybersecurity management.

To further evaluate the FDA's broader efforts to strengthen medical device cybersecurity, reports, white papers, and newsletters published by the FDA's Center for Devices and Radiological Health (CDRH) were examined. Additionally, reports from the United States Government Accountability Office (GAO) to Congressional committees, articles published by the American Hospital Association, and peer-reviewed literature indexed in the National Library of Medicine (NLM) were used for contextual and interpretative purposes only and were not analyzed as primary data.

## 3 FDA cybersecurity safety communications (2013–2025)

As outlined in Table 1, between June 2013 and January 2025, the U.S. FDA issued 18 cybersecurity communications identifying vulnerabilities across various healthcare infrastructures, including medical devices, operating systems, and network servers. While the FDA has confirmed that no patient injuries or fatalities have been reported, the vulnerabilities identified were such that they allowed unauthorized remote access to device systems, exposing the systems to risks like remote code execution, denial of service, device malfunctions, data breaches, and overall system compromise (2, 3). Due to the potential impact on patient safety, device functionality, and data integrity, most of these vulnerabilities were classified as high severity, with each safety communication stipulating specific mitigation recommendations for healthcare providers, patients, and caregivers. A systematic examination of each safety alert is presented below, detailing the vulnerabilities, their impact, and the FDA's suggested remedies.

**Table 1:** List of safety communications and associated cybersecurity vulnerabilities in medical devices.

SL. No:	Date of safety communication	Healthcare Infrastructure at risk	Identified Cybersecurity vulnerability	Severity of the vulnerability
1	13-Jun-13	Network-connected/configured medical devices and hospital networks	Configurable system vulnerabilities and poor security practices	Moderate to High
2	13-May-15	Hospira's LifeCare Infusion Pump Systems	Unauthorized remote access to device systems	High
3	09-Jan-17	St. Jude Medical's Implantable Cardiac Devices and Merlin@home Transmitter	Unauthorized remote access to device systems	High
4	29-Aug-17	Abbott's (formerly St. Jude Medical's) Implantable Cardiac Pacemakers	Unauthorized remote access to device systems	High
5	17-Apr-18	St. Jude Medical implantable cardioverter defibrillator (ICD) and cardiac resynchronization therapy defibrillator (CRT-D)	Configurable embedded computer systems vulnerable to cybersecurity intrusions	High
6	11-Oct-18	Medtronic's cardiac implantable electrophysiology device (CIED)	Unverified Virtual Private Network (VPN) connection that allows unauthorized device access	High
7	21-Mar-19	Medtronic's ICDs and CRT-Ds	Unencrypted Conexus protocol that allows unauthorized device access and manipulation	High
8	27-Jun-19	Medtronic MiniMed™ insulin pumps	Unauthorized remote access to device systems	High
9	01-Oct-19	Real Time Operating Systems (RTOS) used by over 2 billion devices	Unauthorized remote access to device systems, denial of service condition, logical errors and information leaks	High
10	23-Jan-20	GE Healthcare Clinical Information Central Stations and Telemetry Servers	Remote unauthorized access to device systems and connected patient monitors	High
11	03-Mar-20	Medical devices with Bluetooth Low Energy (BLE)	Unauthorized remote access to device systems	High
12	17-Aug-21	Medical devices and supporting systems using older versions of BlackBerry QNX RTOS.	Unauthorized remote access to device systems, denial of service condition	High
13	17-Dec-21	Medical devices and supporting systems using vulnerable versions of the Apache Log4j library.	Remote code execution and unauthorized access to device systems	High
14	22-Dec-21	Fresenius Kabi Agilia Connect Infusion Systems	Remote exploitation, enabling unauthorized access to device systems	High
15	08-Mar-22	Medical devices using PTC Axeda agent and Axeda Desktop Server	Unauthorized remote access to device systems	High
16	02-Jun-22	Illumina NextSeq 550Dx and other related Illumina next-generation sequencing (NGS) instruments	Unauthorized access to the software, potentially impacting patient results and compromising customer networks	High
17	20-Sep-22	Medtronic MiniMed Insulin Pump System	Unauthorized access to device systems	High
18	30-Jan-25	Patient monitoring systems	Unauthorized access to critical patient data or device functionality	High

Source: U.S. Food and Drug Administration. (2025, January 30). Cybersecurity safety communications and other alerts. https://www.fda.gov/medical-devices/digital-health-center-excellence/cybersecurity#safety.

### 3.1 US FDA safety communications (2013–2017)

In June 2013, the FDA issued a safety communication in relation to vulnerability of medical devices containing configurable embedded computer systems which enable connection to the internet, hospital networks, or other devices such as tablets and smartphones. These network-connected medical devices were found to be susceptible to malware infections, potentially compromising devices that managed patient data or controlled monitoring systems. Key vulnerabilities included the use of hard-coded passwords, delayed security updates, and weaknesses in off-the-shelf software. In response, the FDA recommended that manufacturers implement strong authentication protocols, avoid hard-coded passwords, ensure timely deployment of security patches, and incorporate fail-safe modes to maintain device functionality during a breach. The FDA further advised healthcare facilities to strengthen network security by restricting unauthorized access, maintaining up-to-date antivirus software, and monitoring network activity for potential threats (5).

In May 2015, an FDA safety alert was issued in response to cybersecurity vulnerabilities identified in Hospira's Lifecare infusion pump systems (PCA3 and PCA5), which are used to administer therapeutic or anesthetic drugs continuously through a remotely programmable system. These vulnerabilities could allow adversaries to manipulate the pump settings, potentially resulting in the over-infusion or under-infusion of critical medications. The FDA recommended that healthcare facilities perform a thorough risk assessment, follow the risk mitigation measures provided by Hospira, such as isolating the pump systems from the internet and untrusted networks, and adhere to the cybersecurity practices outlined in the FDA's 2013 Safety Communication (6).

The FDA safety communication release in January 2017 addressed vulnerabilities in St. Jude Medical's implantable cardiac devices (ICDs) and the corresponding Merlin@home Transmitter. The ICDs are designed to manage heart rhythm disorders, while the transmitter wirelessly transmits device data to physicians. Identified vulnerabilities in the transmitter allowed unauthorized access to a patient's implanted device, potentially altering the device's programming, causing battery depletion or inappropriate pacing/shocks. To mitigate these risks, St. Jude Medical released a software patch, which was automatically applied to the device. Patients were also advised to keep the transmitter connected to the Merlin.net network for updates. The FDA also recommended continued in-office follow-ups and adherence to manufacturer instructions for ongoing care (7).

In August 2017, the FDA issued a safety communication regarding Abbott's (formerly St. Jude Medical's) implantable cardiac pacemakers, which were susceptible to unauthorized access by malicious actors. If manipulated, it could allow an attacker to alter the pacemaker's programming, potentially administering inappropriate pacing. These devices had been implanted in approximately 465,000 patients, and reports confirmed the possibility of cybersecurity intrusions and exploitation. In response, St. Jude developed a firmware update that required any device attempting to communicate with the implanted pacemakers to be authorized. The FDA approved the update and recommended that healthcare providers discuss the associated risks and benefits of the update with patients, and ensure that device settings and data were printed or digitally stored to prevent loss of date during the update process (8).

### 3.2 US FDA safety communications (2018–2020)

In April 2018, the FDA issued a safety communication regarding Abbott's (formerly St. Jude Medical) RF-enabled Implantable Cardioverter Defibrillators (ICDs) and Cardiac Resynchronization Therapy Defibrillators (CRT-Ds). These devices are used to treat heart conditions such as bradycardia, where the heart rate is abnormally low and tachycardia, where the heart rate is abnormally fast. The communication followed reports of rapid battery depletion, caused by lithium clusters, which created abnormal electrical connections, preventing the device from delivering life-saving pacing or shocks, and potentially leading to patient death. A firmware update was approved to issue vibratory alerts if rapid battery drain is detected, notifying the user of the issue. The update also addressed cybersecurity vulnerabilities in these devices, which could allow unauthorized access to the system, enabling attackers to modify device settings. For devices unable to accept the update, an option to disable RF communication was provided.

In October 2018, the FDA issued a safety communication about cybersecurity vulnerabilities in Medtronic CareLink and CareLink Encore Programmers (models 2090 and 29901), used to update software in Medtronic CIEDs. The software is typically downloaded via the Medtronic Software Distribution Network (SDN) or through a USB plugged into the programmer by a Medtronic representative. The issue stemmed from the programmers not verifying their Virtual Private Network (VPN) connection before downloading updates, allowing unauthorized users to access device systems and alter its functionality. In response, on October 5, 2018, the FDA approved a Medtronic update that blocks the affected programmers from accessing the SDN and recommended that adverse events be promptly reported to better address the vulnerabilities.

In March 2019, an FDA safety alert was issued regarding cybersecurity vulnerabilities in the wireless telemetry technology used for communication between Medtronic's implantable cardiac devices, such as ICDs and CRT-Ds, the Medtronic CareLink Programmers and home monitors. The wireless telemetry system relies on radio frequency (RF) communication between devices, enabling remote monitoring and allowing clinicians to adjust the settings of the implanted devices. However, the telemetry system lacked encryption, authentication, and authorization mechanisms which allowed unauthorized individuals to intercept or manipulate the communication. To mitigate these risks, the alert advises patients to operate the Medtronic programmers within well-managed, secure IT networks and to use only home monitors that are obtained from healthcare providers (9).

In June 2019, the FDA issued a safety communication concerning cybersecurity risks in Medtronic MiniMed™ insulin pumps, which are wearable devices that deliver a continuous supply of insulin, helping people with diabetes manage their blood sugar levels. It was found that the vulnerabilities in the pumps' cybersecurity could allow an unauthorized individual to alter pump settings, potentially causing hypoglycemia ((a medical condition due to excessive insulin delivery) or ketoacidosis (a condition resulting from the interruption of insulin delivery). Since no updates to the affected pumps could mitigate the cybersecurity risk, 11 models of the pumps were recalled. The alert instructed patients to keep the insulin pump and any connected devices within their control while awaiting a replacement. It also emphasized not sharing the pump's serial number, staying alert to pump notifications, alarms, and alerts, and regularly monitoring glucose levels (10).

In October 2019, the FDA issued a safety communication pertaining to 11 vulnerabilities, termed as URGENT/11, which posed risks to certain medical devices and hospital networks. Discovered by a security firm, it was found that these vulnerabilities may allow an unauthorized individual to remotely take control of a device, alter its function, causing data leaks, denial of service, or device malfunctions. The alert also highlighted vulnerabilities in IPnet, a third-party software used in many medical devices, some of which are still in use despite no longer being supported by the original vendor. In response, the FDA recommended that manufacturers collaborate with operating system vendors to implement patches, use unaffected operating systems, and monitor network traffic for exploitation signs (11).

In January 2020, the FDA issued a safety alert in response to cybersecurity vulnerabilities in GE Healthcare Clinical Information Central Stations and telemetry servers used for monitoring patient data. The vulnerabilities could allow an attacker to gain control of the device, disrupt the system, or potentially cause malfunctions in the alarm systems of connected patient monitors. It was found that the attack may go undetected, as it could be mistaken for normal network communication. To minimize these risks, the FDA recommended segregating the networks connecting patient monitors from hospital networks and using VPNs and firewalls, to reduce the likelihood of remote or local network attacks. A software patch is promised by GE Healthcare to address these vulnerabilities (12).

In March 2020, the FDA issued a safety communication to alert the public about 12 vulnerabilities, collectively known as SweynTooth, associated with Bluetooth Low Energy (BLE) wireless communication technology. BLE, through its pairing functionality, enables two devices to exchange information while performing their intended functions. These vulnerabilities identified could allow unauthorized users to crash the device, bypass security measures, or access restricted settings. The FDA recommended that manufacturers of devices using BLE assess the risks, monitor medical devices for any unusual behaviour, and develop software patches to mitigate these risks (13).

### 3.3 US FDA safety communications (2021–January 2025)

In August 2021, the FDA issued a safety communication following BlackBerry's disclosure that its QNX Real-Time Operating System (RTOS) was affected by the BadAlloc vulnerability, impacting multiple RTOSs and associated components. The vulnerabilities could potentially allow an adversary to gain unauthorized access to device systems, cause a denial of service, or even execute malicious code on the affected device. Approximately thirteen BlackBerry QNX products were found to be vulnerable. In response, the Cybersecurity and Infrastructure Security Agency (CISA) has directed that QNX-based systems apply patches for the affected products. In cases where no patch is available, manufacturers are advised to implement the recommended mitigation measures (14).

In December 2021, the FDA issued a safety communication following the identification of critical cybersecurity vulnerabilities within Apache's Log4j software, specifically a serious remote code execution (RCE) flaw. Log4j is extensively used across both consumer and enterprise services, including medical devices, where it ensures system integrity and monitoring. The vulnerabilities presented a significant risk, as they could allow attackers to remotely exploit the flaw, gaining unauthorized access to affected devices and potentially rendering the systems completely inoperable, compromising its overall performance and security. Given the gravity of these vulnerabilities, it was recommended that all product and service maintainers using Log4j thoroughly assess and patch their systems, using updates from Apache or trusted sources (15).

On December 22, 2021, the FDA issed a safety communication regarding vulnerabilities in the Fresenius Kabi Agilia Connect Infusion System, which enables users to control various Agilia infusion pumps, including tools to remotely update and configure the pumps through the Fresenius Kabi Centerium server. A total of 13 vulnerabilities were discovered across multiple components of the Agilia Connect Infusion System, including the infusion pump's Wi-Fi module and software. These vulnerabilities were remotely exploitable, allowing unauthorized access to sensitive data, alteration of device settings, and potential malicious actions, as though they were an authenticated user. In response, Fresenius Kabi released software updates to address these issues, while hardware modifications were required for approximately 1,200 infusion pumps. Until the hardware upgrades are completed, temporary defensive measures were recommended, and healthcare organizations were advised to follow the guidance from CISA and Fresenius Kabi, to mitigate the cybersecurity risks (16).

In March 2022, the FDA issued a safety alert pertaining to vulnerabilities in the Axeda Agent remote connectivity software and Axeda Desktop Server, known as the Access:7 vulnerabilities. These vulnerabilities affected the software's ability to securely enable remote desktop access via the internet and affected various medical devices, Internet of Things (IoT) devices, and other embedded systems that depend on the vulnerable product. It was determined that all versions of the software, used as part of a cloud-based IoT platform, are impacted and if exploited, the vulnerabilities could allow an unauthorized user to fully compromise the host operating system, leading to remote code execution, unauthorized alteration of configurations, and denial-of-service conditions. PTC, the company that owns these web-based technologies, has recommended that manufacturers update to newer software versions, use unique passwords, remove installation files, and restrict remote server access to trusted hosts (17).

In June 2022, the FDA issued a safety communication regarding cybersecurity vulnerabilities in certain Illumina's next generation sequencing instruments. These instruments, used for clinical diagnostics and genetic research, were found to have vulnerabilities in their Local Run Manager (LRM) software. The malicious actors could remotely control the instruments, altering settings or data, potentially leading to incorrect results or data breaches. In response, Illumina developed a software patch to mitigate the vulnerability and is actively working on a permanent software fix for all affected instruments. Additionally, the FDA recommended that healthcare providers promptly install the patches and remain vigilant in reporting any adverse events (18).

In September 2022, the FDA issued a safety alert regarding cybersecurity vulnerabilities in certain Medtronic MiniMed insulin pumps and related components, including the continuous glucose monitor (CGM) transmitter and the CareLink USB device. While it is stated that exploiting these vulnerabilities would require a malicious actor to be within the range of the wireless signal and likely possess technical expertise, reports indicate that these vulnerabilities create a scenario in which an attacker could pair with the system components and gain unauthorized access to the device, potentially compromising the pump and resulting in the delivery of an incorrect insulin dose. In response, Medtronic recommended that users stay vigilant by closely monitoring pump notifications and ensuring that connected system components are managed by an authenticated user. Users were also advised to avoid sharing critical device details and to continuously monitor blood glucose levels (19).

The most recent U.S. FDA safety alert issued in January 2025, was related to cybersecurity vulnerabilities in certain patient monitors used in healthcare and home settings to evaluate vital signs, such as heart rate, blood pressure, and temperature, through remote or central monitoring systems. These vulnerabilities allowed unauthorized users to remotely take control of the devices, perform undesirable actions, such as enabling the collection of personally identifiable information (PII) and protected health information (PHI). A backdoor was discovered in the monitor software, which could allow adversaries to bypass cybersecurity controls, potentially affecting the device's functionality and patient safety. The FDA recommended that healthcare providers assess whether their monitors are affected, switch to local monitoring features, and disconnect Ethernet cables, or disable wireless capabilities until a patch is available. Users were also urged to stop using remote monitoring if their device relied on it (20).

## 4 Key findings

### 4.1 Severity of vulnerability

**Figure 1:**
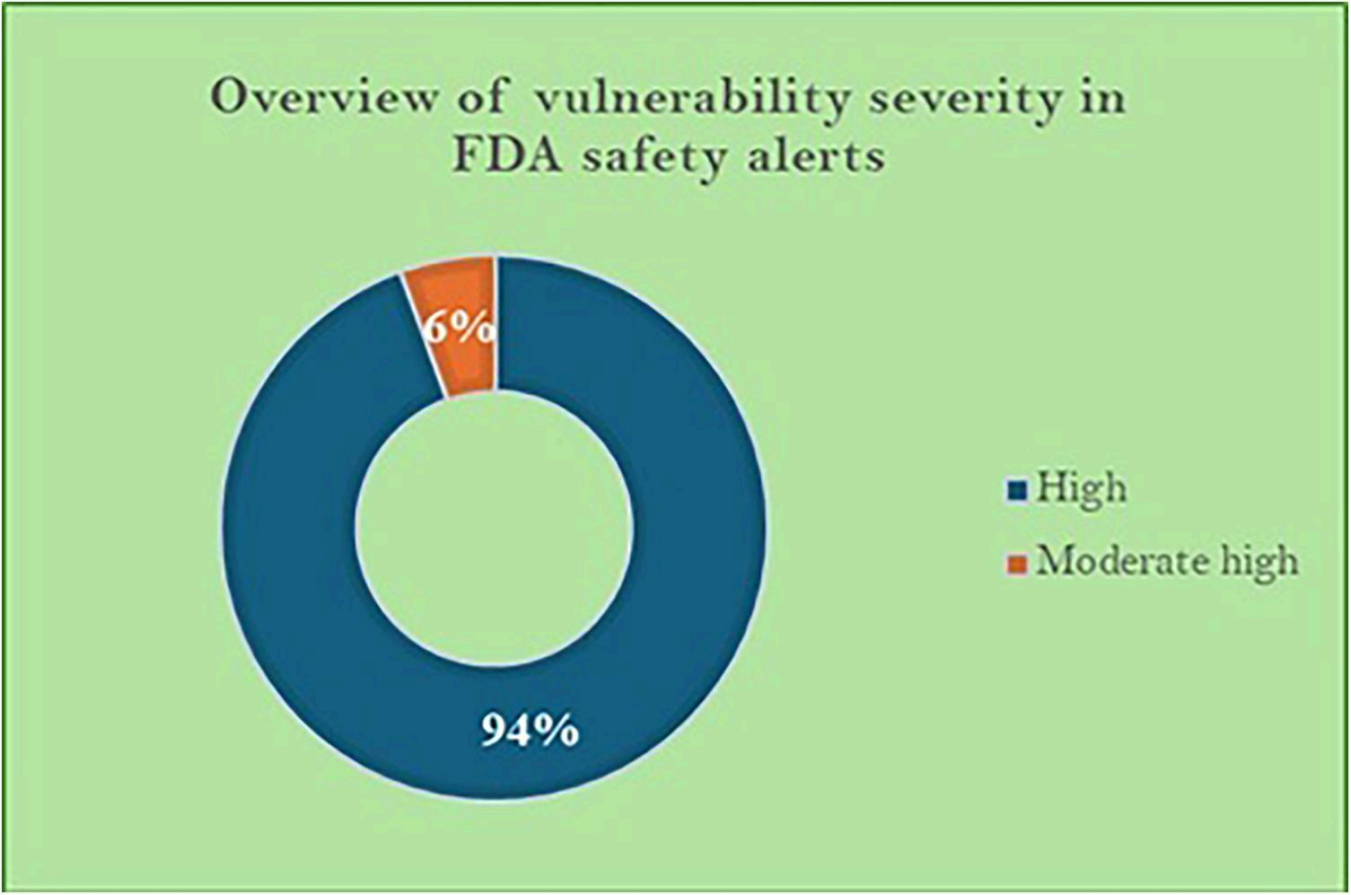
Overview of vulnerability severity in FDA safety alerts.

Between June 2013 and January 2025, the FDA issued 18 safety communications about various cybersecurity vulnerabilities identified across multiple healthcare infrastructures, particularly medical devices, operating systems, and network servers. An analysis of these safety alerts, as shown in Figure 1, reveals that approximately 94% of the vulnerabilities were classified as high-risk. This is particularly concerning because they allowed unauthorized remote access to device systems, posing risks such as device malfunctions, potential remote code execution, and data breaches.

### 4.2 Surge in cybersecurity alerts

**Figure 2:**
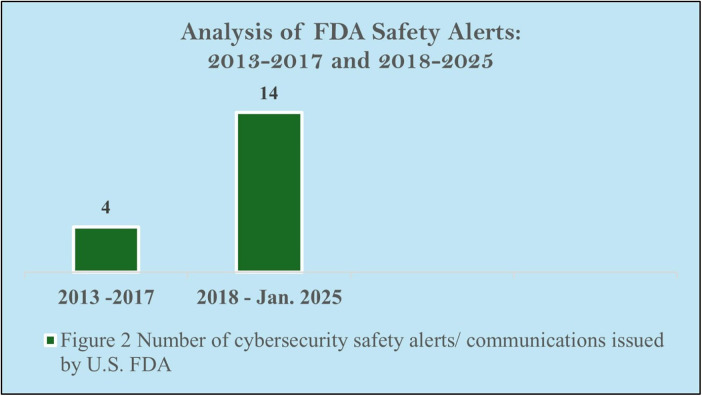
Analysis of FDA safety alerts: 2013–2017 and 2018–2025.

As illustrated in Figure 2, the number of FDA cybersecurity alerts has notably increased from 4 between 2013 and 2017 to 14 between 2018 and 2025. This substantial rise emphasizes the growing prevalence of cybersecurity vulnerabilities in medical devices and healthcare systems. This trend signals the urgent need for enhanced security protocols and comprehensive strategies to tackle emerging risks, necessitating a thorough reassessment of current cybersecurity practices to safeguard patient safety and device integrity in the future.

### 4.3 Healthcare infrastructure at risk

**Figure 3:**
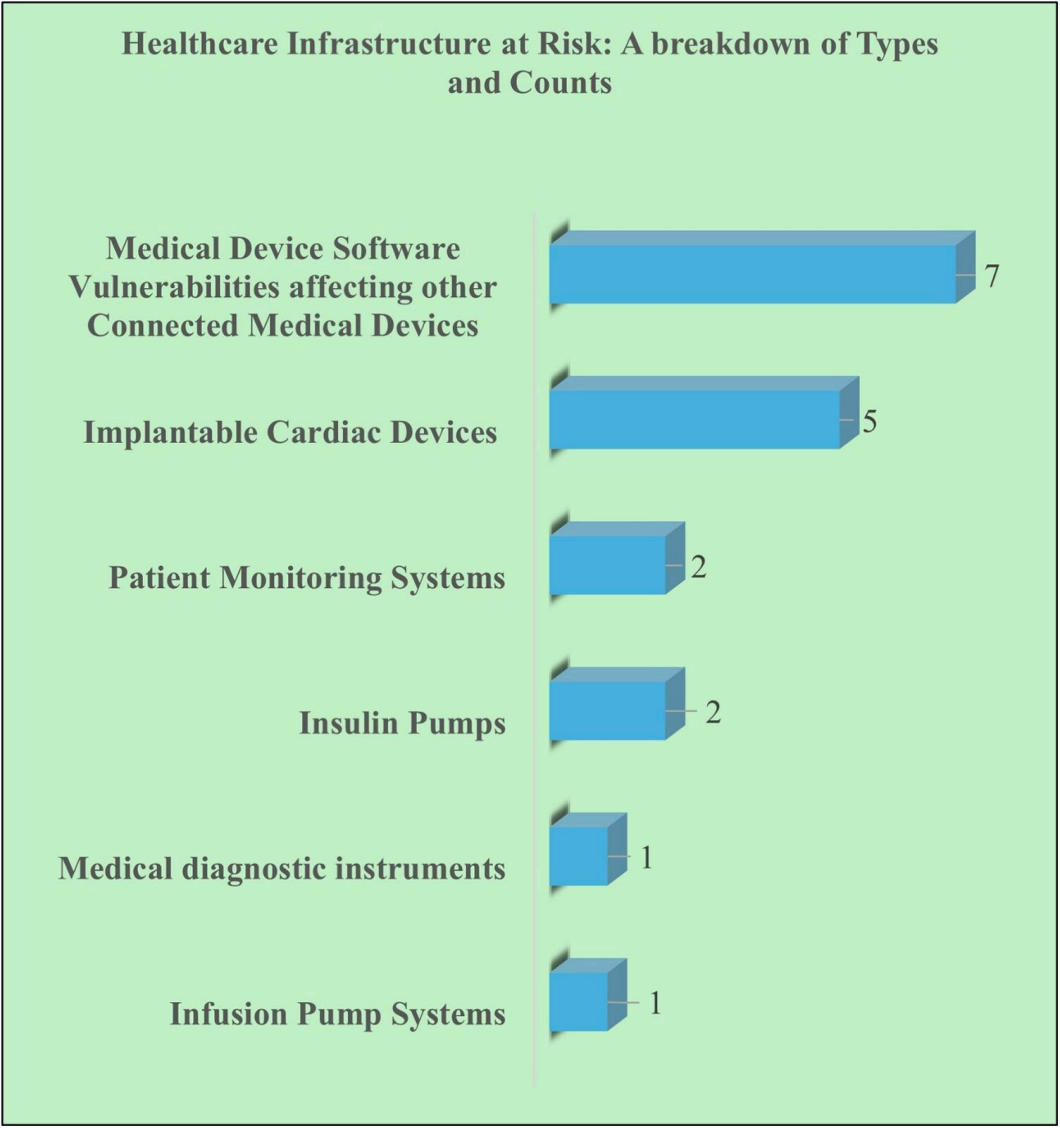
Healthcare infrastructure at risk: a breakdown of types and counts.

As outlined in Figure 3, a total of eighteen FDA safety alerts were issued in relation to vulnerabilities in medical devices and systems, encompassing both software and hardware-related issues that impact critical aspects of patient care. Of these, seven alerts were linked to medical device software vulnerabilities that compromise essential functions, including device control, real-time data collection and visualization, seamless data transfer and integration across healthcare platforms, and the ability to perform firmware updates and remote maintenance to incorporate safety features and bug fixes.

Five alerts were associated with cybersecurity vulnerabilities in implantable medical devices, such as pacemakers and defibrillators, which are responsible for regulating heart rhythms or delivering electrical shocks, to prevent or treat arrhythmias.

Three alerts concerned non-surgically inserted external devices like infusion pumps and insulin pumps, which administer fluids, medications, or insulin at precise intervals to manage chronic conditions, with vulnerabilities posing risks to critical treatment delivery.

Two alerts were linked to patient monitoring systems, which track and display real-time vital signs, enabling healthcare professionals to monitor patient trends and take timely, informed actions to ensure optimal care.

One alert was related to vulnerabilities in medical diagnostic instruments, which are crucial for diagnosing diseases, assessing disease progression, and guiding treatment plans to support clinical decisions.

### 3.4 Key vulnerabilities identified in medical devices and healthcare systems and its consequential impact on patient care and safety

**Figure 4:**
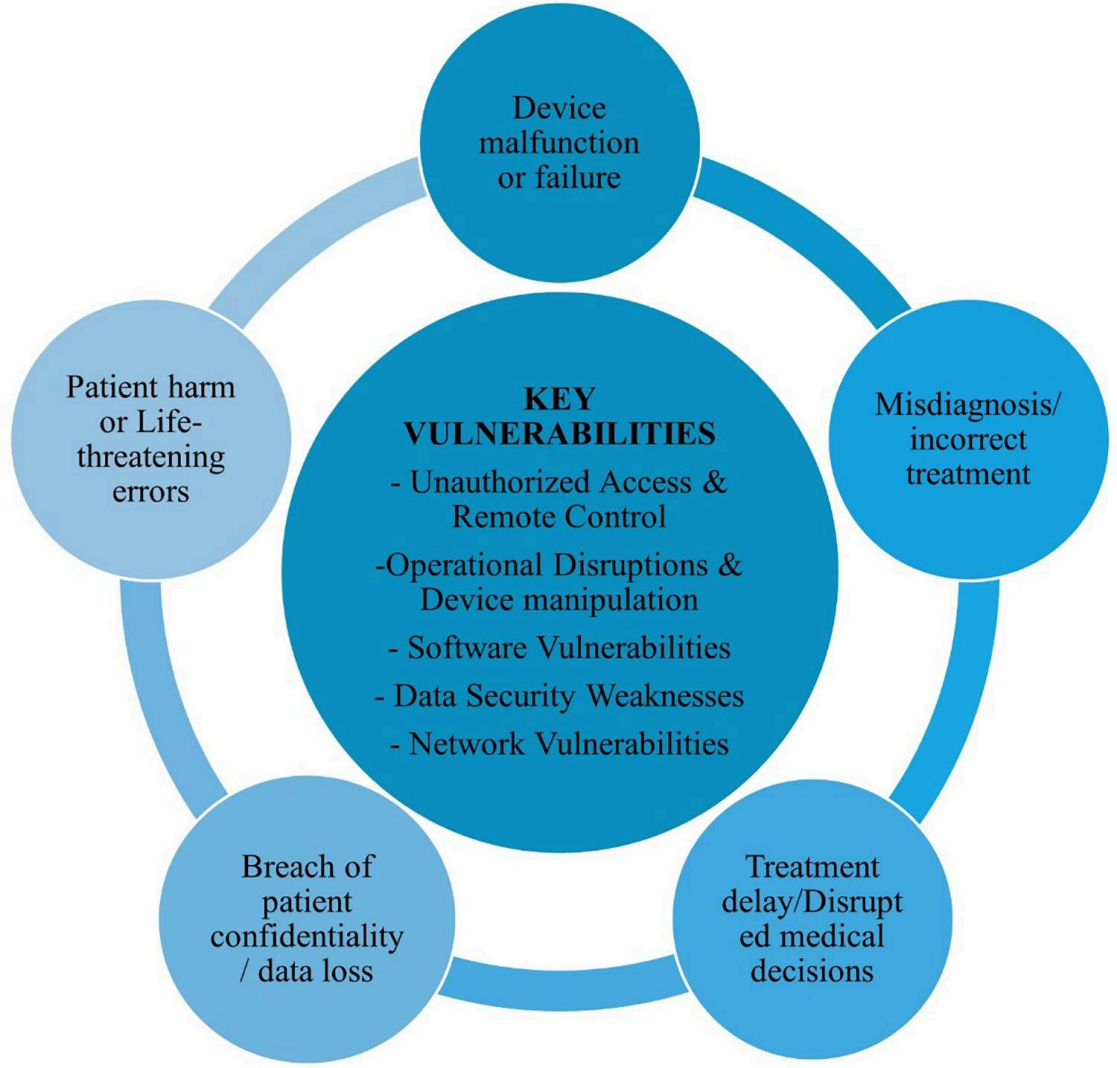
Key vulnerabilities identified in medical devices and healthcare systems and its consequential impact on patient care and safety.

As detailed in Figure 4, the key vulnerabilities identified in the US FDA safety communications involved instances where unauthorized users or malicious actors could gain access or remotely control medical devices. These vulnerabilities included operational disruptions, device manipulation, software flaws, data security weaknesses, and network vulnerabilities. The alerts also pointed out that the potential consequences of these vulnerabilities could result in device malfunction or failure, misdiagnosis or incorrect treatment, treatment delays or disrupted medical decisions, breaches of confidentiality and data loss, as well as patient harm or life-threatening errors.

## 5 Discussion

The analysis of FDA cybersecurity safety communications over the period 2013–2025 highlights several important trends and implications for medical device safety, healthcare infrastructure, and regulatory oversight. The growing frequency of safety communications demonstrates that cybersecurity vulnerabilities have emerged as a prominent concern for medical devices. The surge in alerts, particularly after 2018, suggests that as devices integrate more wireless connectivity features, the potential for high-impact vulnerabilities escalates.

Additionally, the predominance of high-severity vulnerabilities across diverse device types indicates that these weaknesses are not isolated incidents but systemic challenges inherent to modern medical technology. The consequences of exploitation extend to data breaches and potential compromises in device functionality and patient safety, emphasizing the critical need for proactive risk management strategies. These findings establish that cybersecurity is now an essential component of medical device safety rather than a peripheral technical concern. Therefore, there is a pressing need to integrate standardized cybersecurity frameworks, implement continuous risk assessment, and adopt proactive monitoring across pre-market and post-market regulatory processes.

Finally, FDA communications suggest that a collaborative, shared-responsibility approach is essential. Manufacturers, healthcare providers, and patients must coordinate to address vulnerabilities effectively, particularly in highly networked systems where exploitation could have cascading effects. Strengthening such collaboration, along with adopting rigorous cybersecurity practices, is crucial to mitigate the risks and maintain integrity of healthcare delivery in an increasingly interconnected digital health environment.

Third, FDA communications illustrate a regulatory approach that balances immediate risk mitigation with interim measures such as firmware updates, restricted connectivity, and enhanced monitoring.

## 6 Limitations of the study

The study is limited to a qualitative analysis of FDA-issued cybersecurity safety communications and does not incorporate direct perspectives from medical device manufacturers, clinicians, or patients. Consequently, the findings reflect regulatory risk communication rather than stakeholder experiences or operational responses. In addition, the anlysis does not apply quantitative risk assessment methods or formal cybersecurity scoring frameworks, as the study focuses on qualitative examination of regulatory communications and patient safety implications. Future research could strengthen these findings by integrating stakeholder perspectives, quantitative risk modelling, or established cybersecurity frameworks.

## 7 Conclusion

The U.S. FDA safety communications serve as a critical reminder that, as technological advancements continue to expand the potential and capabilities of medical devices, there is a growing surge in the number of vulnerabilities associated with their interconnectedness. These risks extend beyond device failures, threatening entire healthcare systems and potentially causing irreparable harm to patients. What was once a distant concern is now a pressing reality, as safety alerts expose the cybersecurity threats within these devices. While innovations like Elon Musk's Neuralink, which aims to treat paralysis by allowing patients to control devices with their minds, illustrate the remarkable progress in medical technology, they also expose the sector to new cybersecurity challenges that could compromise patient care and data security.

In response to these escalating risks, the FDA has taken a leading role in safeguarding the cybersecurity of medical devices. Recognizing that effective security can only be achieved through heightened awareness and the mandatory implementation of fundamental cybersecurity measures, the FDA issued its final guidance in September 2023, titled *Cybersecurity in Medical Devices: Quality System Considerations and Content of Premarket Submissions.* This guidance provides essential recommendations for addressing cybersecurity concerns in medical devices and specifies the required information for premarket submissions. Additionally, in December 2016, the FDA released guidance focused on the post-market management of cybersecurity vulnerabilities in medical devices, emphasizing a risk-based approach for reporting such vulnerabilities (21). Recent legislative amendments, such as Section 3305 of the Consolidated Appropriations Act of 2023, strengthen cybersecurity requirements for medical device manufacturers. These provisions mandate that manufacturers develop plans for monitoring and addressing cybersecurity risks, disclose any vulnerabilities, and implement robust security protocols for both devices and associated systems. Moreover, initiatives like incident response and threat modeling playbooks aim to better equip healthcare organizations to prepare for and manage cybersecurity incidents effectively (22).

As digital health technologies rapidly advance, the FDA's safety alerts emphasize the critical need for ongoing vigilance. While the guidance highlights the importance of updates, it lacks specific timelines and enforcement for timely patch implementation, potentially delaying the resolution of critical security flaws and leaving devices vulnerable to threats for extended periods. Additionally, another concern is the insufficient focus on securing the device supply chain, as the guidance does not fully hold manufacturers accountable for ensuring the cybersecurity of third-party components, which can become vulnerable entry points for cyberattacks and compromise the overall security of the device. Although cybersecurity threats can never be fully eradicated, mitigating these risks demands a united effort between manufacturers and healthcare providers to ensure strict compliance during the approval process and prompt action in the post-market phase (23). By strengthening cybersecurity protocols, fostering effective collaboration, and ensuring continuous monitoring, medical devices can be more effectively protected in an increasingly complex and interconnected healthcare system.

## Data Availability

The original contributions presented in the study are included in the article/Supplementary Material, further inquiries can be directed to the corresponding author.
